# Rising incidence trends of synchronous prostate and rectal cancers: a population-based study

**DOI:** 10.2340/1651-226X.2025.42592

**Published:** 2025-03-07

**Authors:** Elias Edfelt, Mehrnoosh Shahrivar, Karin Holmsten, Cecilia Radkiewicz

**Affiliations:** a Upper Gastrointestinal Surgery, Department of Molecular Medicine and Surgery, Karolinska Institutet, Stockholm, Sweden; b Colorectal Surgery, Department of Molecular Medicine and Surgery, Karolinska Institutet, Stockholm, Sweden; c Department of Surgery and Oncology, Capio Sankt Görans Hospital, Stockholm, Sweden; d Department of Oncology and Pathology, Karolinska Institutet, Stockholm, Sweden

**Keywords:** Incidence, multiple primary neoplasms, synchronous, prostate cancer, rectal cancer

## Abstract

**Background:**

There is a lack of comprehensive reports on time trends in synchronous prostate and rectal cancers. To address this, we conducted the largest cohort study to date to assess these trends in a population-based setting.

**Methods:**

We included all adult (ages 18−99) men with incident prostate cancer in the Swedish Cancer Register in 1993−2019. Age-standardized incidence rates (ASIRs) of prostate cancer per 100,000 male population per year were calculated and compared to the ASIR of synchronous (± 6 months from rectal cancer diagnosis) prostate cancer. Age-adjusted synchronous-to-general incidence rate ratios (IRRs) were predicted using Poisson regression. As a sensitivity analysis to assess the effect of incidental findings due to the anatomical proximity, we investigated synchronous prostate and non-sigmoid colon cancers.

**Results:**

Among 238,252 prostate cancer cases, 594 were synchronous with rectal cancer. The incidence of synchronous prostate cancer increased over the study period, with mean ASIR rising from 418/100,000 (1993−2001) to 788/100,000 (year 2011−2019). The synchronous-to-general IRR increased from 1.92 (95% confidence interval (CI) 1.60−2.31) to 2.61 (95% CI 2.32−2.95) over the same periods. Prostate cancer was also more commonly diagnosed in conjunction with non-sigmoid colon cancer than in the overall male population, but no time trend was observed.

**Interpretation:**

The incidence of synchronous prostate and rectal cancers has increased over the past 20 years in Sweden, with no signs of plateauing. Future studies are warranted to explore factors contributing to prostate cancer overdiagnosis and to optimize clinical management strategies for this complex patient group.

## Introduction

Globally, prostate and colorectal cancers rank the second and third most common malignancies in men, respectively [1]. While the incidence of these cancers has been rising over recent decades, the trend (both malignancies) has stabilized in Human Development Index high countries in the past decade [1]. Age and family history are key risk factors for both cancers, whereas lifestyle and dietary factors, such as sedentary behaviors and animal-source foods, are more prominently associated with colorectal cancer [1–3]. Approximately 30% of colorectal tumors are located in the rectum [4]. Pelvic magnetic resonance imaging (MRI) is routinely used for diagnosing and staging both prostate and rectal cancers [5, 6]. Pelvic MRI is however not standard for colon cancer diagnosis, and tumors in the colon, except for distal (borderline rectal) sigmoid tumors, are typically undetected using pelvic MRI. Reports on synchronous prostate and rectal cancer are scarce, and comprehensive, population-based studies assessing long-term trends are lacking [7–12]. Available evidence suggests an increasing incidence of synchronous prostate cancer in men with rectal cancer, likely driven by incidental prostate tumors detected on rectal cancer staging MRI [11].

Synchronous prostate and rectal cancers pose significant clinical challenges. Due to their anatomical proximity, concurrent curative treatments carry a higher risk of locoregional adverse events [13]. Additionally, prior pelvic cancer surgery or radiotherapy complicates the diagnosis, treatment, and outcomes of a second pelvic primary cancer [11, 13–19]. Consensus guidelines for the optimal management of this patient group remain unavailable.

In conclusion, synchronous prostate and rectal cancers represent an understudied and neglected clinical entity. Despite indications of increasing incidence, there is a lack of comprehensive studies using contemporary population-based data. To address this gap, we conducted a Swedish population-based cohort study to quantify and outline trends in synchronous prostate and rectal cancer.

## Material and methods

We included all adult (age 18−99 years) men with a prostate and/or rectal cancer record in the Swedish National Cancer Register (NCR) in years 1993−2019. Double reporting by responsible physician and pathologist is mandatory by Swedish law, granting a high coverage of approximately 96% [20]. Publicly available data on population counts by calendar year and age were retrieved from Statistics Sweden.

**Figure 1:**
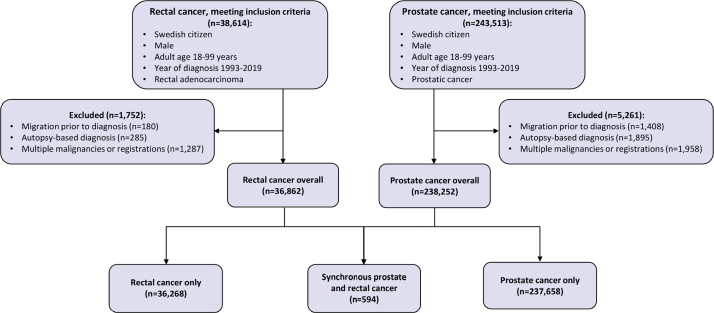
Strengthening the Reporting of Observational Studies in Epidemiology (STROBE) flowchart reporting the number of rectal and prostate cancer patients meeting inclusion criteria, excluded, and included in the final analysis.

Rectal cancer was defined according to the International Classification of Diseases 10th edition (ICD-10) topography code C19, C20, or C21 together with a Systematized Nomenclature of Medicine-Oncology 2nd revision (SNOMED-0/2) histopathology code compatible with adenocarcinoma (Supplementary Table 1). Since prostate cancer is not consistently pathology-verified, it was solely ascertained using the ICD-10 topography code C61. The established International Association of Cancer Registries and International Agency for Research on Cancer definition of synchronous presentation, that is prostate and rectal cancer being diagnosed within 6 months (183 calendar days), was used [21]. See Figure 1 for a Strengthening the Reporting of Observational Studies in Epidemiology (STROBE) flowchart report on the number of prostate and rectal cancer patients meeting inclusion and exclusion criteria.

Numbers and proportions (%) of patients by age at diagnosis (18−49, 50−59, 60−69, 70−79, 80−89, and 90−99 years), calendar period (years 1993−2001, 2002−2010, and 2011−2019), and prostate cancer M (metastasis) stage were calculated by patient group (rectal cancer only, synchronous prostate and rectal cancers, and prostate cancer only). Tumor-node-metastasis (TNM) was introduced in the NCR in year 2004 and reached acceptable coverage in 2008, and the prostate cancer stage was categorized as M0 (localized), M1 (metastasized), and MX (unknown or missing M stage) and restricted to calendar year of diagnosis 2008−2019.

Prostate cancer incidence rates by year were computed as the number of new cases per 100,000 person-years in both the male overall and rectal cancer cohort. Incidence rates were directly age-standardized (ASIR) to the 2019 Swedish male population. Using person-years as the denominator ensured that incidence rates reflect true time-dependent measures, rather than static proportions. The mean ASIR of prostate cancer in the general male population and the mean ASIR of synchronous prostate cancer in the male rectal cancer cohort were calculated by calendar period (years 1993−2001, 2002−2010, and 2011−2019). Time trends were further visualized by plotting yearly empirical ASIR estimates for general and synchronous prostate cancers together with smooth ASIR predictions derived from Poisson regression models with restricted cubic splines (two degrees of freedom) over calendar years 1993−2019.

To quantify the relative (excess) risk of being diagnosed with prostate cancer in conjunction with rectal cancer, we predicted synchronous-to-overall prostate cancer incidence rate ratios (IRRs) with 95% confidence intervals (CIs) adjusted for age and stratified by calendar period, again using Poisson regression. To illustrate time trends, we used Poisson regression with restricted cubic splines (two degrees of freedom), allowing for a flexible time-dependent effect of rectal cancer on prostate cancer risk over the calendar years 1993−2019. While linear models assume a constant rate of change, restricted cubic splines provide flexibility in modeling complex relationships, allowing for a more accurate visualization of real-world patterns without overfitting the data. Increasing the number of degrees of freedom (flexibility) and/or adjusting the placement of knots does not substantially alter neither the smoothed ASIR nor IRR predictions over calendar year.

To distinguish the potential influence of shared risk factors for prostate and colorectal cancers from the effect of incidental cancer detection on pelvic MRI, we conducted a sensitivity analysis. This involved replicating the previously described statistical methods to assess time trends in synchronous prostate and non-sigmoid colon cancer incidence.

This study is reported according to the STROBE checklist for observational studies. It is approved by the Swedish Ethical Review Authority (2020-06617) and conducted in accordance with the Declaration of Helsinki. Statistical tests were two-sided, and the statistical significance level was set to <0.05. All data management and statistical analyses were performed using Stata Intercooled version 17 (StataCorp LP).

## Results

We identified 238,252 cases of prostate and 36,862 cases of rectal cancers, whereof 594 were synchronous (Figure 1 and Table 1). No cases of synchronous prostate cancer were diagnosed in the youngest age-group (18−49 years), and the vast part (44%) occurred in the age interval (70−79 years) when rectal cancer peaks, roughly mimicking the prostate cancer age distribution in the overall male population. The number of synchronous prostate cancer cases increased from 113 (19%) in 1993−2001 to 268 (45%) in 2011−2019. The prostate cancer M stage distribution was similar in men with rectal cancer (synchronous) and overall. Additionally explored variables (rectal cancer anatomical subsite, pathology-verification of prostate adenocarcinoma, prostate tumor stage, and rectal tumor stage) were well balanced between groups (Supplementary Table 2). Supplementary Figure 1 shows frequency counts of synchronous prostate and rectal cancer cases over days (-183 to 183) between dates of diagnoses, and 42% (*n* = 252) of synchronous cases were diagnosed with prostate cancer first.

**Table 1:** Baseline characteristics presented as numbers (*n*) and percentages (%) of Swedish adult (age 18–99 years) men diagnosed with rectal cancer only, synchronous prostate and rectal cancer, or prostate cancer only, in calendar years 1993–2019.

All study participants (*n* = 274,264)	Rectal cancer	Synchronous	Prostate cancer
	Numbers (%)	Numbers (%)	Numbers (%)
	36,268 (100)	594 (100)	237,658 (100)
Age (years)		
18–49	1,799 (5)	0 (0)	1,907 (1)
50–59	4,383 (12)	25 (4)	25,600 (11)
60–69	10,043 (28)	178 (30)	83,458 (35)
70–79	12,717 (35)	261 (44)	85,804 (36)
80–89	6,656 (18)	123 (21)	37,958 (16)
90–99	670 (2)	7 (1)	2,931 (1)
Calendar period		
1993–2001	10,073 (28)	113 (19)	57,987 (24)
2002–2010	12,250 (34)	213 (36)	85,897 (36)
2011–2019	13,945 (38)	268 (45)	93,774 (40)
Prostate cancer stage*		
M0	N/A	259 (76)	89,579 (78)
M1	N/A	23 (7)	6,573 (6)
MX	N/A	57 (17)	18,317 (16)

* Subgroup analysis including calendar year of diagnosis 2008–2019.

The prostate synchronous with rectal cancer ASIR increased from 418 (in 1993−2001) to 788/100,000 person-years (in 2011−2019) corresponding to synchronous-to-overall age-adjusted IRR of 1.92 (95% CI 1.60−2.31) and 2.61 (95% CI 2.32−2.95, Table 2). In the same time interval, prostate cancer synchronous with non-sigmoid colon cancer was increased compared to prostate cancer overall but showed no trend of increase over calendar time, with an IRR of 1.83 (95% CI 1.52−2.21) in 1993−2001 and 1.63 (95% CI 1.41−1.88) in 2011−2019.

**Table 2:** Prostate cancer overall, synchronous with rectal, and synchronous with non-sigmoid colon cancer age-standardized incidence rate (ASIR)/100,000 person-years and synchronous-to-overall age-adjusted incidence rate ratios (IRRs) including 95% confidence intervals (CIs) by calendar period.

Calendar period	Prostate cancer overall		Prostate cancer synchronous with rectal cancer		Prostate cancer synchronous with non-sigmoid colon cancer	
	ASIR	IRR	ASIR	IRR (95% CI)	ASIR	IRR (95% CI)
1993–2001	228	ref.	418	1.92 (1.60–2.31)	373	1.83 (1.52–2.21)
2002–2010	298	ref.	647	2.19 (1.92–2.51)	442	1.43 (1.22–1.69)
2011–2019	273	ref.	788	2.61 (2.32–2.95)	455	1.63 (1.41–1.88)

ASIR: Age-standardized incidence rates; IRR: incidence rate ratios; CI: Confidence intervals; ref: reference.

**Figure 2:**
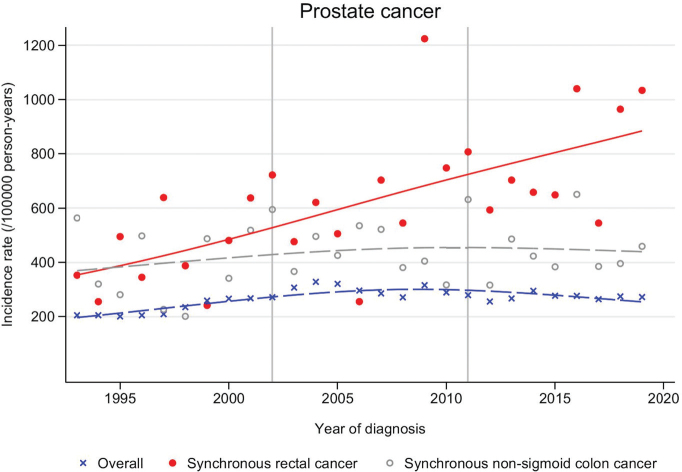
Prostate cancer age-standardized incidence rate/100,000 person-years over calendar year of diagnosis 1993–2019, overall (all Swedish men), synchronous with rectal cancer, and synchronous with non-sigmoid colon cancer, empirical estimates (dots) and smoothed predictions (lines).

Prostate cancer ASIRs (overall, synchronous with rectal cancer and synchronous with non-sigmoid colon cancer) were plotted over calendar years 1993−2019 (Figure 2). Prostate in conjunction with rectal cancer increased steadily throughout the study period while prostate cancer overall increased slightly up until around year 2005 and thereafter stabilized. Prostate cancer synchronous with non-sigmoid colon cancer ASIR was modestly but consistently elevated compared to prostate cancer overall but otherwise followed the prostate cancer overall calendar time pattern.

**Figure 3:**
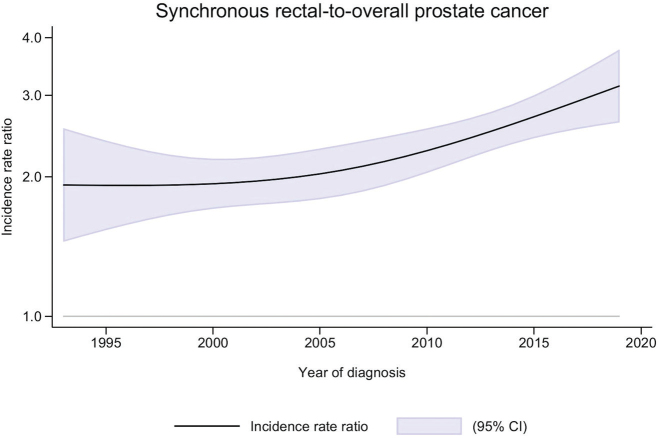
Age-adjusted incidence rate ratio (IRR) of synchronous-to-overall prostate cancer, including 95% confidence interval (CI, represented by the shaded area) over calendar years of diagnosis 1993–2019.

The prostate cancer synchronous with rectal-to-overall IRR over calendar years 1993−2019 (Figure 3) was statistically significant (95% CI not including 1.00) over the whole time period and increased from around year 2005, without indications of reaching a plateau.

## Discussion

This is the largest and only population-based cohort study to date, investigating trends in synchronous prostate and rectal cancer trends over an extended 27-year period. We observed a substantial and statistically significant increased risk of being diagnosed with prostate cancer in conjunction with rectal cancer, with this risk showing a continuous increase over the past 20 years. While prostate cancer was also more common among men with non-sigmoid colon cancer compared to those without colorectal cancer, no evidence of an increasing trend was found.

Our study has several limitations. First, both rectal and prostate cancers may be linked to modifiable factors such as sedentary behavior and obesity, information that was unavailable [2, 22]. Second, the observational design and the lack of individual-level information on MRI utilization prevent us from establishing a causal relationship between pelvic MRI use and the detection of synchronous prostate and rectal cancers. Additionally, our data sources lack critical clinical details, like prostate cancer stage, Gleason score, and PSA, limiting inferences on potential detection bias and overdiagnosis of clinically insignificant prostate tumors. PSA testing was implemented in standard care before the study period and thus should not affect the observed time trends. Third, the register-based approach carries a risk of information bias, as reporting to the NCR may have improved over time, potentially contributing to the observed increase in incidence. We consider this to be non-differential, as any reporting changes would likely affect both synchronous and general prostate cancer cases equally. A further potential source of bias is the misdiagnosis of a locally advanced rectal cancer as prostate cancer, or vice versa, due to direct invasion between the two tumors. Given the rarity of direct invasion, we believe this to be of minor importance [23]. Finally, underreporting of elderly patients may introduce uncertainty in the age-incidence patterns but is unlikely to differentially affect synchronous and general prostate cancers [20].

The strengths of our study include the unprecedented size and long study period, which enabled clinically relevant analyses of trends in synchronous prostate and rectal cancers over time. Furthermore, the nationwide, population-based design with near-complete coverage reduces selection bias, making our results generalizable to other settings with similar sociodemographic compositions and healthcare systems.

Existing literature is limited, characterized by small sample sizes [9, 24], single-institution settings [8, 24], and a focus on reported proportions rather than incidence rates [11]. Moreover, discrepancies in the definitions of ‘synchronous’ cancer further complicate comparisons. For instance, two studies defined synchrony as occurring simultaneously or within 3 months [11, 24], while another allowed for a timeframe of up to 12 months [7]. This wide variation in study methodologies and definitions makes it challenging to directly compare findings across studies and with the results of the present investigation.

The observed increase in synchronous prostate and rectal cancer incidences over recent years can be attributed to several plausible factors. A major contributor is likely the enhanced utilization of pelvic MRI in clinical practice. Sturludottír et al. found that the most common reason for a synchronous prostate cancer diagnosis was incidental findings on rectal cancer staging MRI [11]. In Sweden, MRI for rectal cancer staging became widely adopted around 2005 and was incorporated into national guidelines by 2008 [11]. For prostate cancer, MRI use began to increase in the mid-2010s and was officially recommended as a first-line diagnostic tool prior to prostate-biopsy in 2020 [25]. This shift has likely facilitated the detection of synchronous malignancies, as reflected in our findings. Specifically, we observed a stable synchronous-to-general prostate cancer IRR of approximately 2.0 in the first 10 years of the study period, followed by a notable increase after the turn of the millennium. This trend aligns with the growing use of MRI in rectal cancer staging and suggests that the rise in the IRR is further driven by the increasing adoption of MRI as a standard diagnostic tool for prostate cancer [5, 6].

The excess risk of synchronous prostate and non-sigmoidal colon cancers was smaller and did not increase over the study time period. Shared risk factors and/or symptomatology likely partly explain the excess risk of synchronous prostate cancer in conjunction with colorectal cancer. Obesity, defined as a body mass index of ≥ 30 kg/m^2^, has increased among Swedish men over the study period and is associated with various cancers, including an elevated risk of developing secondary primary malignancies [26–28]. While the link between obesity and rectal cancer is well established, evidence connecting obesity to prostate cancer remains unclear [3, 28]. When patients present with bowel or urinary symptoms related to their primary cancer, they typically undergo digital rectal exam, which may reveal both prostate and lower rectal tumors. The most likely explanation for the baseline excess risk of prostate cancer in both rectal and colon cancer patients is detection bias, leading to the identification of asymptomatic and clinically silent secondary tumors, even before pelvic MRI became a standard diagnostic tool.

In summary, this population-based observational cohort study reports rising incidence rates of synchronous prostate and rectal cancers, but not of synchronous prostate and non-sigmoid colon cancers. These findings suggest that non-biological factors, such as evolving clinical practices and increasing detection rates, may play a larger role than biological mechanisms in explaining the observed trends. However, the study lacked individual-level data on MRI utilization, clinical cancer characteristics, and shared risk factors, highlighting the need for further research to better understand the underlying drivers and their implications. Given the increase in synchronous prostate and rectal cancer incidence, additional studies are crucial to identify optimal diagnostic and treatment strategies for this overlooked patient population.

## Author contributions

Concept and design: EE, MS, KH, and CR. Acquisition of data: CR. Statistical analysis: EE and CR. Analysis and interpretation of data: EE, MS, KH, and CR. Drafting the manuscript: EE and MS. Critical revision of the manuscript for important intellectual content: EE, MS, KH, and CR. Obtaining funding: CR.

## Supplementary Material

Rising incidence trends of synchronous prostate and rectal cancers: a population-based study

## Data Availability

Aggregated data underlying this article can be shared upon request to the corresponding author.
