# Supplementary material for: Rising incidence trends of synchronous prostate and rectal cancers: a population-based study
Source: Acta Oncol. 2025 Mar 7;64:42592. doi: 10.2340/1651-226X.2025.42592 (PMC11905149; doi:10.2340/1651-226X.2025.42592)

Supplementary material has been published as submitted. It has not been copyedited, or typeset by Acta Oncologica

Supplementary Table 1. International classification of diseases 10th revision (ICD-10) topography and Systematized Nomenclature of Medicine-Oncology 2nd revision (SNOMED-O/2) morphology definitions.

	ICD-10		SNOMED-O/2	
Rectal cancer	Rectosigmoid	C19	High grade dysplasia	84801, 84802, 81402/b, 82632, 82112, 82612
	Rectal	C20	Adenocarcinoma	81403, 85603, 80103, 80203, 85103, 84803, 84903
	Anal	C21	Tumour syndromes	82203, 82200/b
Non-sigmoid colon cancer	Caecum	C18.0	High grade dysplasia	84801, 84802, 81402/b, 82632, 82112, 82612
	Appendix	C18.1	Adenocarcinoma	81403, 85603, 80103, 80203, 85103, 84803, 84903
	Ascendens	C18.2	Tumour syndromes	82203, 82200/b
	Right flexure	C18.3		
	Transverse	C18.4		
	Left flexure	C18.5		
	Descendens	C18.6		
	Familial polyposis	C18.8		
	Unspecified	C18.9		
Prostate cancer		C61	N/A	

Supplementary Figure 1. Frequency counts of synchronous prostate and rectal cancer cases over days (- 183 to 183) between dates of diagnoses, left half bars represent cases where rectal cancer was diagnosed first and right half staples cases where prostate cancer was diagnosed first.

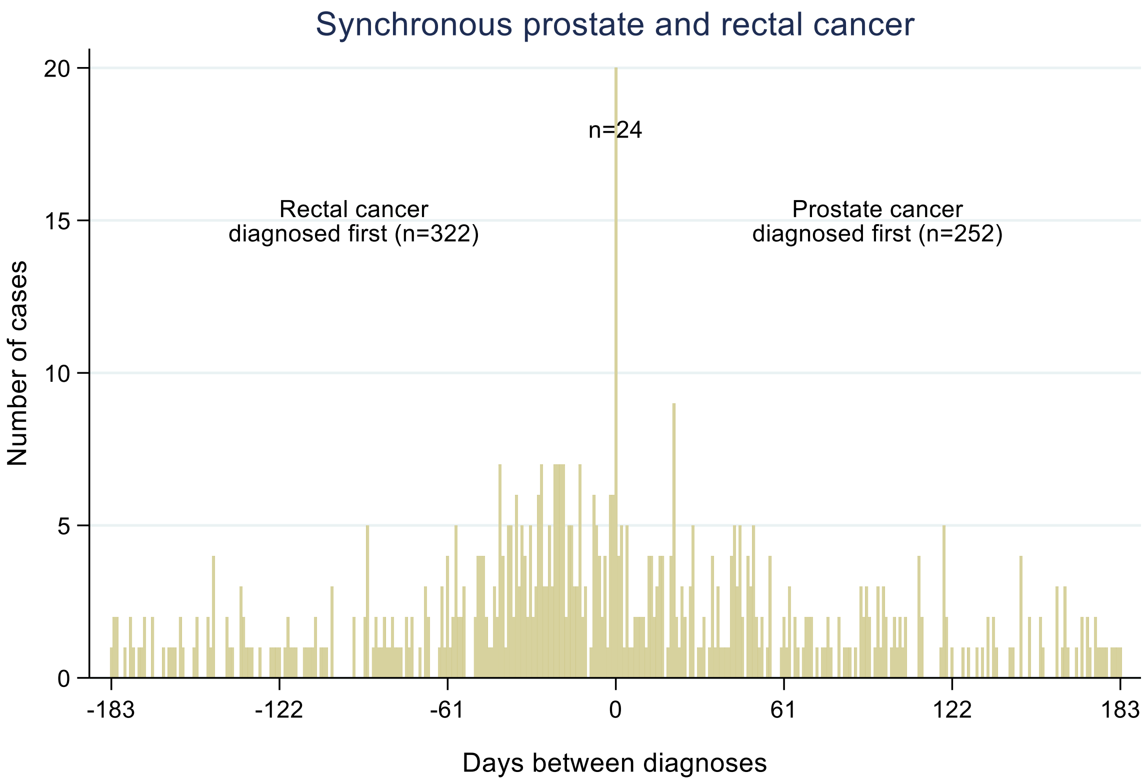

Supplement: Rising incidence trends of synchronous prostate and rectal cancers: a population-based study [file AO-64-42592-s1.pdf]
